# Morphological Evolution of Hybrid Block Copolymer Particles: Toward Magnetic Responsive Particles

**DOI:** 10.3390/polym15183689

**Published:** 2023-09-07

**Authors:** Jaeman J. Shin

**Affiliations:** 1 Department of Materials Science and Engineering, Soongsil University, Seoul 06978, Republic of Korea; jshin@ssu.ac.kr; 2 Department of Green Chemistry and Materials Engineering, Soongsil University, Seoul 06978, Republic of Korea

**Keywords:** block copolymer particle, hybrid, magnetic nanoparticle, gold nanoparticle

## Abstract

The co-assembly of block copolymers (BCPs) and inorganic nanoparticles (NPs) under emulsion confinement allows facile access to hybrid polymeric colloids with controlled hierarchical structures. Here, the effect of inorganic NPs on the structure of the hybrid BCP particles and the local distribution of NPs are studied, with a particular focus on comparing Au and Fe_3_O_4_ NPs. To focus on the effect of the NP core, Au and Fe_3_O_4_ NPs stabilized with oleyl ligands were synthesized, having a comparable diameter and grafting density. The confined co-assembly of symmetric polystyrene-*b*-poly(1,4-butadiene) (PS-*b*-PB) BCPs and NPs in evaporative emulsions resulted in particles with various morphologies including striped ellipsoids, onion-like particles, and their intermediates. The major difference in PS-*b*-PB/Au and PS-*b*-PB/Fe_3_O_4_ particles was found in the distribution of NPs inside the particles that affected the overall particle morphology. Au NPs were selectively localized inside PB domains with random distributions regardless of the particle morphology. Above the critical volume fraction, however, Au NPs induced the morphological transition of onion-like particles into ellipsoids by acting as an NP surfactant. For PS-*b*-PB/Fe_3_O_4_ ellipsoids, Fe_3_O_4_ NPs clustered and segregated to the particle/surrounding interface of the ellipsoids even at a low volume fraction, while Fe_3_O_4_ NPs were selectively localized in the middle of PB domains in a string-like pattern for PS-*b*-PB/Fe_3_O_4_ onion-like particles.

## 1. Introduction

Block copolymer (BCP) particles have received great attention due to their facile preparation process for generating nanostructured polymer colloids via confined self-assembly in evaporative emulsion [1,2,3]. Recent advance in this field has been driven by numerous works on achieving precise control over the particle morphologies and shapes by engineering the emulsion interface using surfactants [4,5,6,7,8], and, thus, affording a diverse plethora of BCP particle shapes including prolate, oblate, cone, and patchy surfaces via the spontaneous deformation of the particle/surrounding interface [9,10]. These particles have widespread potential applications including catalyst support [11,12,13], photonic materials [14,15,16], and stimuli-responsive materials for biomedical applications [17,18,19,20,21]. For implementing these particles into the applications mentioned above, however, functionalization with inorganic materials can assist in attaining the required property of interest. Therefore, numerous investigations have been performed on the co-assembly of BCPs and inorganic nanoparticles (NPs) under emulsion confinement [22]. These studies include a fundamental understanding of the co-assembly behavior of BCPs and NPs that determines the local distribution and orientation of NPs in the BCP matrix [4,23,24,25,26], which in turn allows facile access to BCP/NP hybrid particles with a controlled structure and corresponding optical [27], electronic [28], and magnetic [29] properties.

Despite extensive studies summarized above, Au NPs have been a predominant choice for studying the co-assembly behavior of BCP and NPs, presumably due to their stability and ease of functionalization with various organic/polymeric ligands. On the other hand, magnetic NPs exhibit important physical characteristics that have led to applications including MRI contrast agents [30,31], material recycling [32], and orientation-dependent photonic properties [33]. Nevertheless, fundamental studies on the spatial distribution of magnetic nanoparticles inside the microphase-separated BCP particles have been less reported. Unfortunately, examples of the magnetic functionalization of the polymeric colloids focus on simpler compartmentalized particles, such as the addition of iron oxide NPs into the one face of the Janus particle prepared via the macrophase separation of two homopolymers, with the application as a recoverable Pickering emulsion surfactant [34,35,36]. Other examples include the use of polystyrene-*b*-poly(acrylic acid) (PS-*b*-PAA) BCPs as a tool for coating the surface of magnetic nanoparticles for inducing macrophase separation with polystyrene-*b*-poly(dimethylsiloxane) (PS-*b*-PDMS) BCPs to obtain shape-anisotropic particles [37,38].

Here, the morphological evolution of hybrid BCP particles is investigated based on the co-assembly of polystyrene-*b*-poly(1,4-butadiene) (PS-*b*-PB) BCPs and NPs under emulsion confinement. Au and Fe_3_O_4_ NPs stabilized by oleic ligands were employed to perform a comparative study on the influence of the inorganic NP core, to provide design rules for preparing nanostructured colloids functionalized with magnetic NPs. In our previous study, a confined assembly of pristine PS-*b*-PB BCP particles resulted in tunable morphologies, including striped ellipsoids, onion-like particles, and their intermediate structures, upon carefully controlling the evaporation rate of the solvent [39]. Building onto this study, introducing Au NPs into the BCP-containing emulsion resulted in the selective localization of Au NPs inside PB domains regardless of the particle morphologies. Also, increasing the amount of Au NPs showed that they can act as an NP surfactant above a critical volume fraction, to allow a morphological transition from onions to ellipsoids regardless of the solvent evaporation condition. On the other hand, the co-assembly of Fe_3_O_4_ NPs and PS-*b*-PB in evaporative emulsion showed different behavior depending on the particle morphologies. For ellipsoidal particles, Fe_3_O_4_ NPs were clustered and segregated to the particle/surrounding interface, while Fe_3_O_4_ NPs were selectively incorporated inside the PB domains for onion-like particles. Furthermore, closer observation of the distribution of NPs inside the BCP domains revealed that Au NPs were well-dispersed within PB domains with edge-filling behavior, whereas Fe_3_O_4_ NPs incorporated into the PB domains formed string-like aggregation at the center of the PB domains.

## 2. Experimental Procedure

### 2.1. Materials

PS_112k_-*b*-PB_104k_ copolymers (*M*_n_ of PS block = 112 kg/mol, *M*_n_ of PB block = 104 kg/mol, dispersity (*Ð*) = 1.06) were purchased from Polymer Source Inc., Dorval, QC, Canada. HAuCl_4_·3H_2_O (99%), oleylamine (OAm, technical grade), tetralin (technical grade), the *tert*-butylamine-borane complex (TBAB), iron (III) acetylacetonate, oleic acid, 1-octadecene (ODE, technical grade), and sodium dodecyl sulfate (SDS) were purchased from Sigma Aldrich (Burlington, MA, USA) and used as received. All commercial-grade organic solvents were purchased from Samchun Chemical (Seoul, Korea) and used as received.

### 2.2. Synthesis of Au NPs

Au NPs were synthesized using a previously reported method [40]. An orange precursor solution containing HAuCl_4_·3H_2_O (150 mg), OAm (15 mL), and tetralin (15 mL) was prepared in the air at room temperature and stirred under nitrogen flow for 10 min. A reducing solution of TBAB (130 mg), OAm (2.5 mL), and tetralin (2.5 mL) was prepared and injected into the precursor solution. The reduction was initiated instantaneously and the solution changed to a deep purple color within 5 s. The mixture was left to react for 1 h before hexane (60 mL) was added to precipitate the Au NPs. The Au NPs were collected by repeated centrifugation (7000 rcf, 5 min) and washing with ethanol three times, followed by re-dispersion in toluene at a designated concentration for use.

### 2.3. Synthesis of Iron Oxide NPs

Iron oxide NPs were synthesized by a previously reported method [41]. A total of 12 mmol of iron (III) acetylacetonate, 100 mmol of oleic acid, 112 mmol of oleyl amine, and 72 mL of 1-octadecene were mixed in a 250 mL flask. The reaction mixture was heated to 110 °C and maintained under vacuum for 2 h. Then, the temperature was increased to 300 °C at a rate of 10 °C/min. After 1 h, the reaction mixture was cooled to room temperature, and iron oxide NPs were precipitated by adding isopropanol. Iron oxide NPs were re-dispersed in hexane and washed further using isopropanol three times.

### 2.4. Preparation of PS-b-PB Colloidal Particles by Membrane Emulsification

A solution containing 2 mg/mL of PS_112k_-*b*-PB_104k_ in toluene (3 mL) was emulsified in a continuous phase containing 5 mg/mL SDS in deionized water (DI-water) (60 mL) using a Shirasu porous glass (SPG) membrane device [39,42,43]. Monodisperse emulsion droplets of PS-*b*-PB were generated by passing the organic phase through the membrane with a pore size (*d_pore_*) of 1.1 μm. Subsequent removal of toluene at room temperature resulted in the monodisperse PS-*b*-PB particles. Afterward, the final sample was collected by centrifugation for 5 min at 10,000 rcf and re-dispersed in DI-water. This centrifugation–redispersion process was repeated three times to remove residual surfactants.

### 2.5. Characterizations

Weight fractions of NP ligands were measured by thermogravimetric analysis (TGA) from 20 °C to 600 °C with a heating rate of 20 °C min^−1^. Transmission electron microscopy (TEM, JEOL 2000 FX, 300 kV, Tokyo, Japan) was used to characterize the gold and magnetic NPs, as well as the internal nanostructures of the hybrid particles.

## 3. Results and Discussion

**Figure 1:**
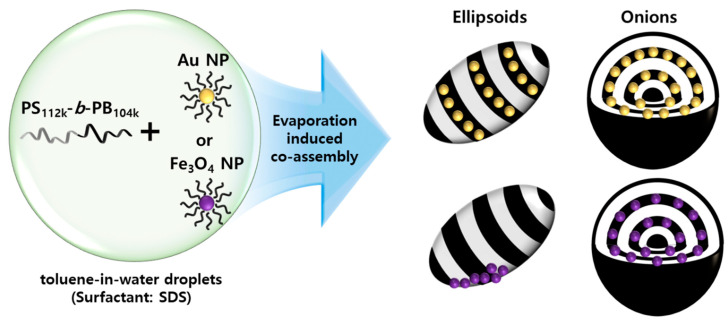
Illustration showing co-assembly of inorganic nanoparticles (Au NP and Fe_3_O_4_ NP) with PS-*b*-PB BCPs by controlled solvent evaporation from toluene-in-water emulsion.

Figure 1 illustrates our experimental system for preparing hybrid BCP particles. After co-dissolving a designated amount of BCPs and NPs (either Au or Fe_3_O_4_) in toluene, the organic phase was emulsified with an aqueous phase containing SDS surfactants using membrane emulsification [39,42,43]. As demonstrated in our previous work, the final morphology of PS-*b*-PB particles is highly affected by the evaporation rate of toluene. The evaporation rate was controlled by tuning the interfacial area between emulsion/air (A_emul/air_) while the emulsion was kept stirring (200 rpm) under ambient conditions (Figure S1a). For the fast evaporation of toluene (A_emul/air_ = 26.4 cm^2^), ellipsoidal particles were generated, while slow evaporation (A_emul/air_ = 0.1 cm^2^) produced onion-like particles [39]. Intermediate morphologies between the ellipsoid and onion-like particles were obtained at A_emul/air_ = 2.3 cm^2^. The size of the particles was strictly controlled with narrow polydispersity, with a long axis = 689.0 ± 78.3 nm and short axis = 414.5 ± 46.8 nm for ellipsoids, and diameters of 520.2 ± 48.6 nm for onion-like particles (Figure S1b,c).

The location and distribution of NPs in the BCP particles are determined by the (i) enthalpic interaction between the NP surface and BCPs, and the (ii) entropic penalty associated with stretching the hosting BCP domains [44,45]. For enthalpic interaction between the NPs and BCPs, control over the surface property of the inorganic NPs by polymeric/organic ligands plays a decisive role in determining the location of NPs within the BCP [23,46,47]. Entropic contributions to the system are determined by the size [48,49], shape [50], and loading amount of the inorganic NPs as well as the molecular weight of the ligand covering the NPs [24,25,51]. Therefore, the size ratio between NPs (*d*) and their associated domain (*L*) is key to determining the spatial distribution of NPs within the BCP matrix. When the size of the NPs is smaller than the radius of gyration of the BCP matrix, NPs can be localized in the hosting domains. When the size of the NPs is larger than the radius of gyration of the BCP matrix, however, the entropic penalty associated with hosting the NPs increases, and NPs are expelled from the hosting domains.

**Figure 2:**
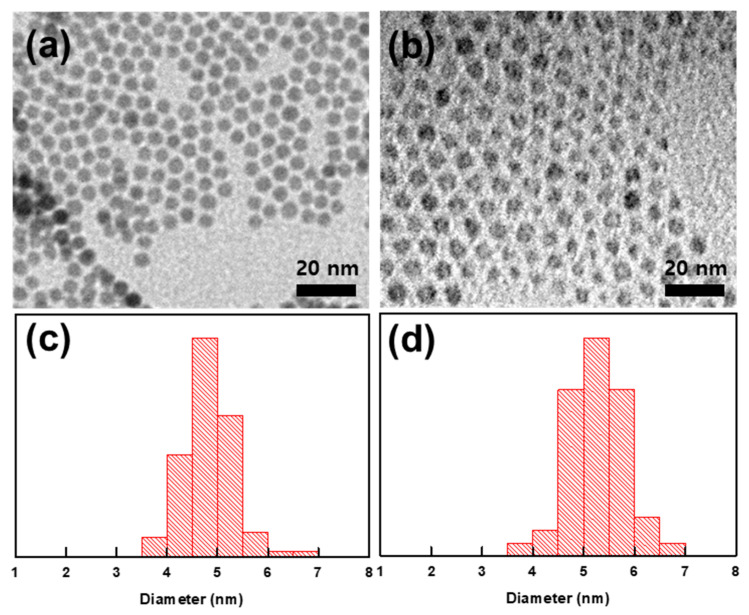
(**a**,**b**) TEM image and (**c**,**d**) size distribution histogram of (**a**,**c**) Au NPs and (**b**,**d**) Fe_3_O_4_ NPs.

To investigate the co-assembly behavior of BCPs and NPs solely as a function of the NP core (Au or Fe_3_O_4_) and exclude additional enthalpic/entropic effects, Au NP and Fe_3_O_4_ NPs with a similar size and surface chemistry were synthesized according to the procedures in previous literature (Figure 2) [40,41]. The surfaces of both NPs were stabilized with oleic ligands, and the surface tension values of the oleyl-functionalized surface, PS, and PB domains were γ_PS_ = 40.7 dyn/cm, γ_PB_ = 30.5 dyn/cm, and γ_octadecene_ = 28.1 dyn/cm (at 20 °C), respectively. The polarities of the two contiguous phases can be calculated through the harmonic mean equation [52,53], thus giving the interfacial tensions of the polymer/NPs as a γ_PS/NP_ = 7.38 dyn/cm and γ_PB/NP_ = 0.33 dyn/cm. Therefore, the interaction between the oleyl-functionalized NP surface and PB domains is much preferred to that of PS, and thus, the localization of NPs within PB domains can be expected upon the co-assembly. Also, NP size was accurately controlled to be similar, with each diameter being 4.8 nm and 5.3 nm for Au and Fe_3_O_4_ NPs, respectively. In this way, the difference in the entropic penalty given by either Au or Fe_3_O_4_ NPs on the host domain of PS-*b*-PB can be minimized. Also, the calculation of ligand grafting density via TGA analysis (Figure S2 and Table S1) showed 6.02 and 4.56 chains/nm^2^ for Au and Fe_3_O_4_ NPs [54]. Despite a small difference, comparable values of grafting density allowed the assumption of a similar enthalpic interaction between the NP surface and the polymer matrix for the Au and Fe_3_O_4_ NPs.

**Figure 3:**
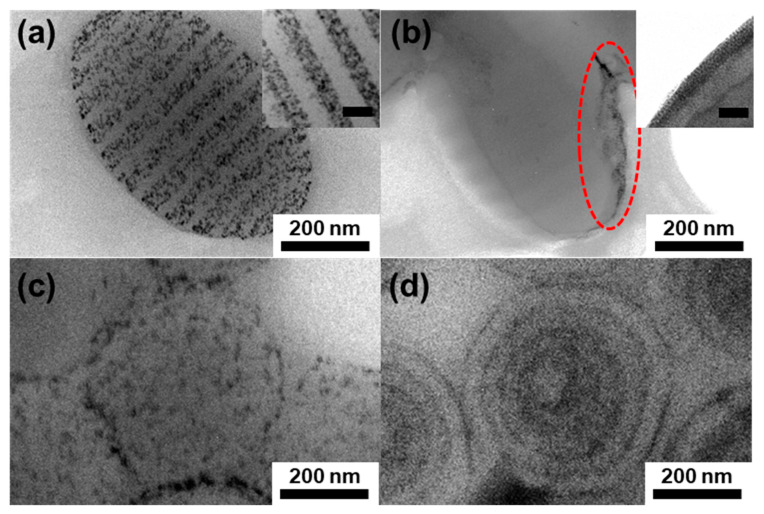
TEM images showing hybrid PS-*b*-PB particles. Striped ellipsoids (**a**,**b**) and onion-like particles (**c**,**d**) containing (**a**,**c**) Au NP or (**b**,**d**) magnetic NPs. Fe_3_O_4_ NPs were segregated at the edge of the ellipsoidal particles as indicated by red dashed circles in Figure 3b. The loading amount of NPs was fixed to 7.0 vol% for both Au and Fe_3_O_4_. Particles were observed without staining. Scale bars in inset figures are 50 nm.

The organization of NPs in hybrid PS-*b*-PB particles was observed using TEM without staining. Figure 3 shows the inner morphologies of the hybrid BCP particles and the NP distribution depending on the particle shape (ellipsoid or onion-like) and the type of NPs (Au or Fe_3_O_4_). For ellipsoidal particles, Au NPs were selectively incorporated into the disk-like PB domains of the ellipsoids (Figure 3a). On the other hand, Fe_3_O_4_ NPs were segregated at the edge of the ellipsoidal particles at the interface between the particle and surrounding aqueous phase, particularly near the pole of the ellipsoid, as indicated by red dashed circles in Figure 3b. For onion-like morphologies, both the Au NPs and Fe_3_O_4_ NPs were well-incorporated into curved PB domains (Figure 3c,d). However, closer observation of the NP distribution inside the PB domains revealed that while Au NPs are randomly distributed within the PB domains, Fe_3_O_4_ NPs were located in the middle of PB domains in a string-like pattern (Figure S3) [55]. Therefore, the concentric lamellae structure of onion-like PS-*b*-PB/Au particles was difficult to recognize using normal TEM, and, rather, confirmed using cross-sectional TEM images (Figure S4). By contrast, concentric lamellae domains were easily distinguishable for the PS-*b*-PB/Fe_3_O_4_-hybrid onion-like particles using normal TEM images (Figure 3d). In addition, the thickness of the concentric layer of NPs was thinner for PS-*b*-PB/Fe_3_O_4_ than that of PS-*b*-PB/Au. These results reflect the fact that Fe_3_O_4_ NPs are concentrated at the center of the PB domains. Considering the same surface chemistry with similar grafting density, it is speculated that an additional driving force should be present for the difference in NP organization, including (i) the aggregation of Fe_3_O_4_ NPs in ellipsoids and (ii) the string-like localization in the middle of PB domains for onion-like particles.

**Figure 4:**
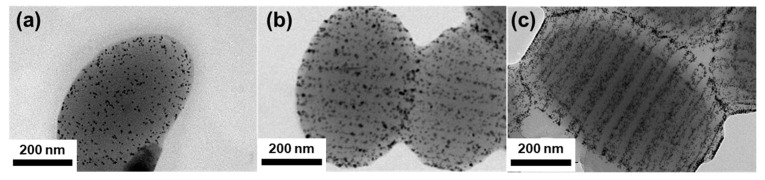
TEM images showing the distribution of Au NPs in PS-*b*-PB/Au ellipsoids as a function of the volume fraction of NP relative to BCP (φ). (**a**) 1.4 vol%, (**b**) 3.5 vol%, and (**c**) 7.0 vol%. Particles were observed without staining. A_emul/air_ = 26.4 cm^2^.

To better investigate the distribution of Au NPs, the morphologies of PS-*b*-PB/Au ellipsoids were observed at different φ values from 1.4 vol%, 3.5 vol%, and 7.0 vol% (Figure 4). For φ = 1.4 vol%, striped lamellae structures were hardly distinguishable based on the distribution of Au NPs (Figure 4a). However, increasing φ to 3.5 vol% showed recognizable striped lamellae structures with Au NPs distributed inside the PB domains, but mainly located at the edge (Figure 4b). Further increasing the φ to 7.0 vol% clearly showed the selective incorporation of Au NPs into PB domains, with additional Au NPs fully covering the whole PB domains (Figure 4c). However, the observation of tilted ellipsoids revealed that the PB lamellae stack is not fully incorporated with Au NPs, and NPs show a ring-like pattern along the PB/surrounding interface (Figure S5). Previous works have shown the importance of the *d*/*L* ratio [56], where the interfacial segregation of NPs in the hosting domain was predicted for NPs with *d*/*L* < 0.2 while localization at the center of the BCP domain was expected for *d*/*L* > 0.3 to reduce conformational entropic penalties for BCP chains accommodating NPs. The *d*/*L* value of our experimental system was 0.15–0.17 (*L_PB_* = 32 nm); thus, the observation in Figure 4 was consistent with the edge-filling behavior of NPs in the BCP domains predicted by the theory.

**Figure 5:**
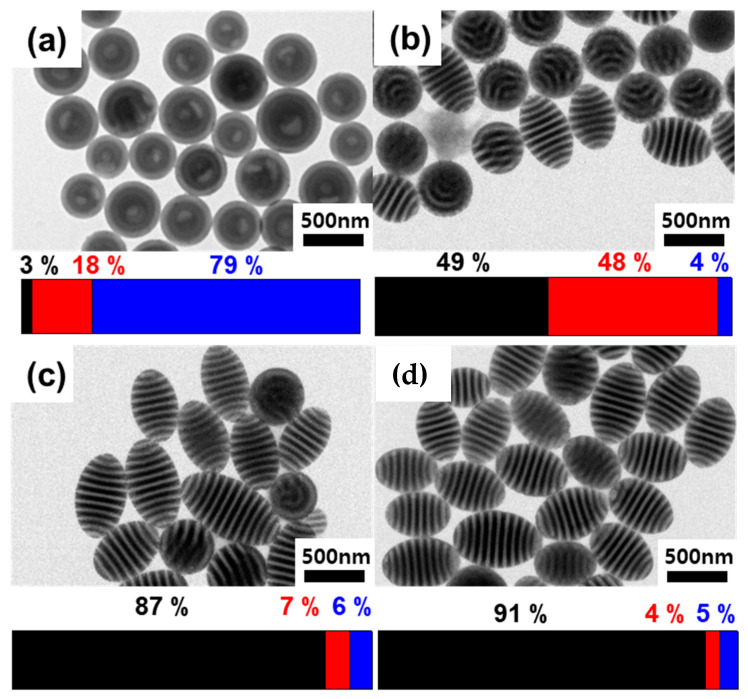
TEM images of hybrid PS-*b*-PB/Au particles as a function of φ. (**a**) Pristine PS-*b*-PB particles, (**b**) φ = 1.4 vol%, (**c**) φ = 3.5 vol%, and (**d**) φ = 7.0 vol%. Each color of the bar chart shows the percentage of each morphology. Black: ellipsoids, red: intermediates, blue: onions. PB domains were stained with OsO_4_. A_emul/air_ = 0.1 cm^2^.

Also, the effect of Au NPs on particle morphologies was investigated. Figure 5 shows the TEM images of hybrid PS-*b*-PB/Au particles and corresponding bar charts showing the frequency of each particle morphologies (Black: ellipsoids, red: intermediates, blue: onions) prepared at slow evaporation conditions (A_emul/air_ = 0.1 cm^2^). The gradual morphological transition from onions to ellipsoids was observed upon the addition of Au NPs. Onion-like particles were observed for pristine PS-*b*-PB without Au NPs (Figure 5a), consistent with the particles generated at a slow evaporation rate [39]. The addition of 1.4 vol% of Au NPs resulted in a mixture of onion-like particles and an intermediate morphology between ellipsoid and onion (Figure 5b). Upon increasing the Au NP content to φ = 3.5 vol% and 7.0 vol%, the majority of particles turned into an ellipsoidal morphology (Figure 5c,d). Overall, hybrid ellipsoidal particles were consistently observed regardless of the solvent evaporation rate (Figure S6) and the particle size (Figure S7) above a critical value of φ.

For φ > 3.5 vol%, a notable portion of Au NPs was positioned at the three-phase interface of the PS/PB/surrounding aqueous phase. This observation shows the possibility of NPs acting as a surfactant, which supports the morphological transition from onion-like particles into ellipsoids observed in Figure 5 [4,7]. For pristine PS-*b*-PB particles, the thermodynamically stable form was the onion-like particles, obtained at A_emul/air_ = 0.1 cm^2^ due to the SDS surfactant showing slight selectivity toward the PB domains [39]. However, once a sufficient amount of Au NPs is positioned at the interface, neutralization of interfacial interaction (γ_PS/surr_~γ_PB/surr_) can occur and ellipsoidal particles decorated with Au NPs can be produced as a thermodynamically stable morphology. The appearance of intermediate morphologies at φ = 1.4 vol% can be understood in a similar context, where 1.4 vol% of Au NPs was not enough to neutralize the particle/surrounding interfacial interaction to result in a mixture of intermediate and ellipsoid morphologies in the single particle batch. One difference from the previous works utilizing Au NP surfactants is that they focused on controlling the size/shape of the NPs to increase the entropic penalty associated with hosting the NPs in the polymeric domains. Therefore, NPs were expelled intentionally to the emulsion surface to act as a surfactant and stabilize shape-anisotropic particles (prolate and oblate) [4,7]. In our system, it is speculated that although Au NPs are incorporated into PB domains, they start to position at the three-phase interface when the edge of the PB domain is saturated with NPs, and this critical point is φ = 3.5 vol%.

**Figure 6:**
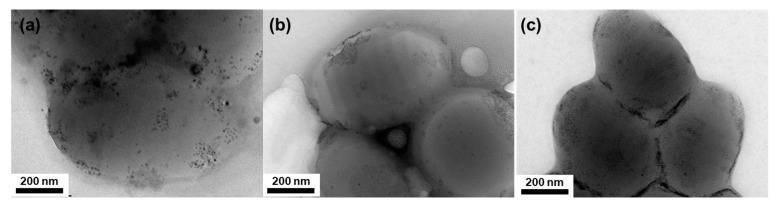
TEM images showing the distribution of Fe_3_O_4_ NPs in PS-*b*-PB striped ellipsoids as a function of φ. (**a**) 1.4 vol%, (**b**) 3.5 vol%, and (**c**) 7.0 vol%. Particles were observed without staining.

On the other hand, the addition of Fe_3_O_4_ NPs did not induce any morphological transitions of the PS-*b*-PB/Fe_3_O_4_ particles. In detail, ellipsoidal particles were obtained at A_emul/air_ = 26.4 cm^2^ and onion-like particles were obtained at A_emul/air_ = 0.1 cm^2^ regardless of the variation in φ (Figure S8), in contrast to the case of Au NPs. To further investigate the distribution of Fe_3_O_4_ NP in the PS-*b*-PB particles, PS-*b*-PB/Fe_3_O_4_ ellipsoids at different φ values from 1.4 vol%, 3.5 vol%, and 7.0 vol% were observed without staining PB domains (Figure 6). For φ = 1.4 vol%, several aggregates of Fe_3_O_4_ NPs were dispersed over the whole particle, having an ellipsoidal shape (Figure 6a). Upon increasing φ to 3.5 vol%, the size of the NP aggregates was larger and mainly located at the interface of the particle/surrounding interface. (Figure 6b). Further increasing the φ to 7.0 vol% showed a similar aggregation of NPs observed both at the low-curvature interface of the particle/surrounding interface and the pole of the ellipsoids (Figure 6c). Fe_3_O_4_ NPs clustered even at a low volume fraction (φ = 1.4 vol%), and a further increase in NP loading led to a larger aggregate at the ellipsoid/surrounding interface. To observe the clustering behavior of NPs during the solvent evaporation, the emulsion was freeze-dried in the middle of the evaporation of toluene and subjected to TEM characterization, which shows that aggregation of Fe_3_O_4_ NPs started at the early stage of solvent evaporation randomly over the particle surface upon increasing the concentration of emulsion via solvent evaporation (Figure S9).

**Figure 7:**
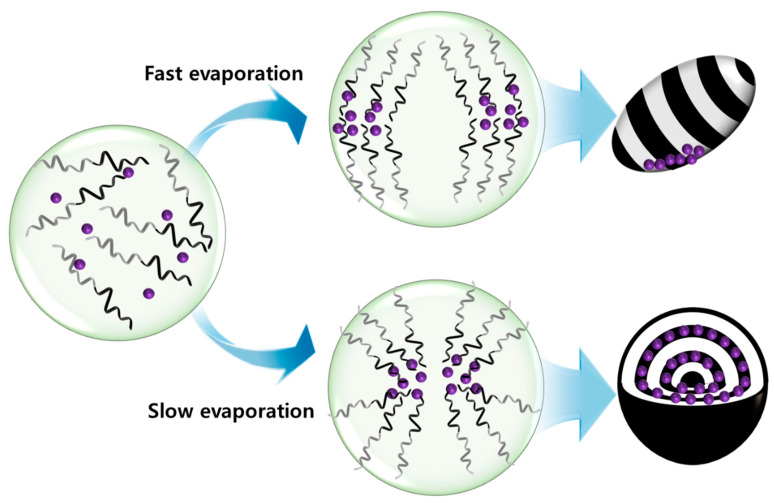
Schematic illustration showing the formation mechanism of PS-*b*-PB/Fe_3_O_4_ ellipsoids and onion-like particles.

Figure 7 illustrates a possible mechanism explaining the difference in the spatial distribution of NPs and subsequent morphological transformations of hybrid PS-*b*-PB particles. While the size of the magnetic NPs used in this study was small, the local aggregation of NPs can enhance the magnetic moment to accelerate the clustering [57,58]. Therefore, the key to the selective localization of NPs in the polymer domains is to suppress the magnetic clustering of NPs. Since toluene is a neutral solvent for PS-*b*-PB, the freely expanded structure of the BCPs is expected in the emulsion droplet, and the incorporation of NPs in the preferred domain can be achieved when the affinity between the NP and solvent is weaker than that between the NP and PB chains [59]. Otherwise, when the affinity between the NP and solvent is stronger, NPs should segregate to the particle/surrounding interface. In our experimental system, Au NPs were successfully localized in the PB domains, indicating that the interaction between oleyl-functionalized NPs and the PB is favored. Also, the selective positioning of Fe_3_O_4_ NPs in the PB domains of onion-like particles can be supported in the same context. The only exception is PS-*b*-PB/Fe_3_O_4_ ellipsoidal particles, in which NPs were clustered and segregated to the particle surface regardless of the loading amounts of NPs.

Consequently, the evaporation rate of toluene was assumed to be the critical parameter determining the aggregation of magnetic NPs. Moon et al. showed that the selectivity of the organic solvent toward BCPs can influence the morphology of the PS-*b*-PI/γ-Fe_2_O_3_ composites [60]. In detail, when the selective solvent was used for casting the BCP/NP composite, BCP micelles were formed that stabilized magnetic NPs inside the core to suppress magnetic clustering, while the use of a neutral solvent for casting resulted in the agglomeration of NPs. In our experimental system, toluene is a near-neutral solvent for PS-*b*-PB but shows slight selectivity toward PS based on the solubility parameters (δ_PS_ = 18.6, δ_PB_ = 17.3, δ_tol_ = 18.2) [61]. Therefore, while BCPs and NPs are freely dissolved inside the toluene droplets, the concentration of BCPs inside the emulsion droplets increases upon the evaporation of toluene and loosely packed micelles can form to encapsulate NPs. Nevertheless, the formation of PS-*b*-PB micelles can be difficult for the fast evaporation of toluene due to insufficient time for micelle formation. Accordingly, the segregation of aggregated Fe_3_O_4_ NPs at the ellipsoid surface can be rationalized by the difficulty of forming BCP micelles during the fast evaporation of toluene (A_emul/air_ = 26.4 cm^2^). By contrast, the successful encapsulation of Fe_3_O_4_ NPs in onion-like particles is attributed to the formation of BCP micelles that encapsulated NPs in the core to suppress the magnetic clustering.

In addition, the confining effect arising from the particle morphology can influence the NP distribution. Wu et al. systematically investigated the effect of the size of magnetic NPs on their dispersion in a styrene–butadiene–styrene (SBS) triblock copolymer matrix [55]. When *d*/*L*~0.5, most magnetic NPs are concentrated in the middle of the PB layers at low particle loading. A similar behavior was observed in our system, where Fe_3_O_4_ NPs aligned in the string-like pattern at the middle of PB domains of the onion-like particles but at a much lower *d*/*L* value of 0.15. Such difference occurs due to the concentric lamellae structure of onion-like particles providing additional conformational strain on the PB chains. Also, magnetic NPs were well-dispersed without aggregation in PS-*b*-PI/NP fiber obtained by electrospinning [62], reflecting the fact that the concentric structure of the BCP matrix can better show its encapsulating properties toward magnetic NPs.

## 4. Conclusions

In conclusion, the co-assembly of BCPs and NPs within emulsion confinement offers a versatile approach to creating hybrid polymeric colloids with various hierarchical structures. This study investigated the influence of inorganic NPs, specifically comparing Au and Fe_3_O_4_ NPs with a similar size and surface chemistry on the resulting structure of hybrid BCP particles and the local distribution of NPs. The co-assembly of symmetric PS-*b*-PB BCPs and NPs in the emulsion yielded a diverse array of particle morphologies, including striped ellipsoids, onion-like particles, and intermediate forms. Notably, a key distinction emerged between PS-*b*-PB/Au and PS-*b*-PB/Fe_3_O_4_ particles in terms of NP distribution within the particles. Au NPs were preferentially localized within PB domains in a random distribution, irrespective of particle morphology. As the NP volume fraction exceeded a critical threshold, morphologies transitioned from onion-like particles into ellipsoidal structures due to the Au NPs effectively serving as NP surfactants. In contrast, Fe_3_O_4_ NPs formed clusters along the interface between the ellipsoid and the surrounding medium, even at low-volume fractions. For PS-*b*-PB/Fe_3_O_4_ onion-like particles, Fe_3_O_4_ NPs were selectively localized within the central region of the PB domains, forming a string-like arrangement. These findings collectively show the importance of controlled NP distribution in shaping the final particle morphology, offering guidelines for the rational design of hybrid nanostructured particles with precisely engineered spatial arrangements of NPs. Accordingly, well-designed particle magnetic NPs show potential applications in magnetic responsive materials, recyclable materials, and magnetically active carriers in the biomedical area.

## Data Availability

Not applicable.

## Supplementary Materials

The following supporting information can be downloaded at: https://www.mdpi.com/article/10.3390/polym15183689/s1, Figure S1: (a) Illustration showing the control of the evaporation rate of toluene by varying the emulsion/air interfacial area (A_emul/air_). Low-magnification TEM image pristine PS-*b*-PB of (a) ellipsoidal particles (A_emul/air_ = 26.4 cm^2^), and (b) onion-like particles (A_emul/air_ = 0.1 cm^2^). Figure S2: TGA traces of (a) Au NPs and (b) Fe_3_O_4_ NPs. Table S1: Physical properties of Au and Fe_3_O_4_ nanoparticles. Figure S3: Schematic illustration showing the difference in the distribution of NPs inside the PB domains. Figure S4: Cross-sectional TEM images of onion-like PS-*b*-PB/Au hybrid particles. Figure S5: TEM images of tilted PS-*b*-PB/Au ellipsoids. φ = 7.0 vol%. Figure S6: Magnified (upper) and low-magnification (lower) TEM images of hybrid PS-*b*-PB/Au striped ellipsoids as a function of solvent evaporation rate. (a) A_emul/air_ = 26.4 cm^2^, (b) A_emul/air_ = 2.3 cm^2^, and (c) A_emul/air_ = 0.1 cm^2^. φ = 7 vol%. Particles were observed without any staining. Figure S7: Magnified (upper) and low-magnification (lower) TEM images of hybrid PS-*b*-PB/Au striped ellipsoids as a function of solvent evaporation rate. Larger-sized ellipsoids were produced by using a 2.1 um SPG membrane. (a) A_emul/air_ = 26.4 cm^2^, (b) A_emul/air_ = 2.3 cm^2^, and (c) A_emul/air_ = 0.1 cm^2^. φ = 7 vol%. Particles were observed without any staining. Figure S8: TEM images of hybrid PS-*b*-PB/Fe_3_O_4_ particles as a function of φ. (a–c) A_emul/air_ = 26.4 cm^2^, (d–f) A_emul/air_ = 0.1 cm^2^. (a,d) pristine PS-*b*-PB particles, (b,e) φ = 1.4 vol%, and (c,f) φ = 3.5 vol%. PB domains were stained with OsO_4_. Figure S9: TEM images of hybrid PS-*b*-PB/Fe_3_O_4_ were obtained by freeze-drying the emulsion after evaporation of toluene for 6 h. A_emul/air_ = 26.4 cm^2^.
