# Supplementary material for: Morphological Evolution of Hybrid Block Copolymer Particles: Toward Magnetic Responsive Particles
Source: Polymers (Basel). 2023 Sep 7;15(18):3689. doi: 10.3390/polym15183689 (PMC10534701; doi:10.3390/polym15183689)
Supplement: Supplementary file 1 [file polymers-15-03689-s001.zip › polymers-2580964-supplementary.pdf]

polymers

Supporting Information

Morphological Evolution of Hybrid Block Copolymer Particles: Toward Magnetic Responsive Particles

Jaeman J. Shin [1,2]

[1] Department of Materials Science and Engineering, Soongsil University, Seoul 06978, Republic of Korea; jshin@ssu.ac.kr
[2] Department of Green Chemistry and Materials Engineering, Soongsil University, Seoul 06978, Republic of Korea

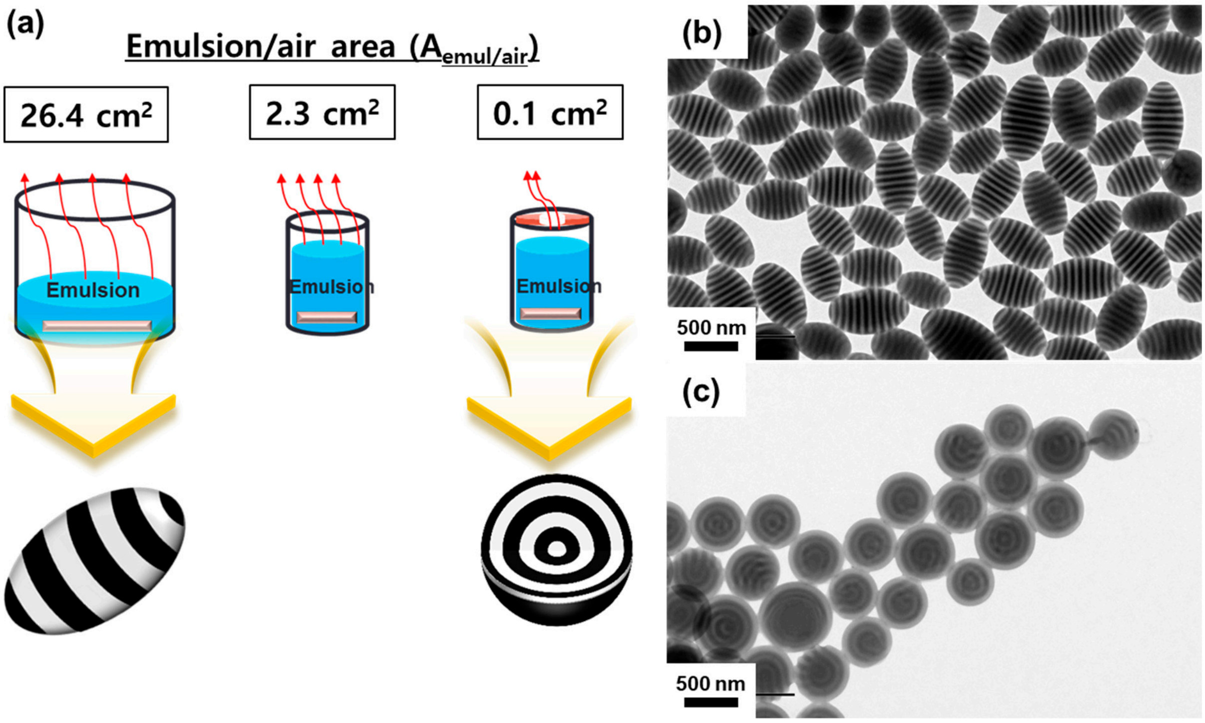

Figure S1. (a) Illustration showing the control of the evaporation rate of toluene by varying the emulsion/air interfacial area ($A_{emul/air}$). Low-magnification TEM image pristine PS-*b*-PB of (a) ellipsoidal particles ($A_{emul/air}$ = 26.4 cm^2), and (b) onion-like particles ($A_{emul/air}$ = 0.1 cm^2).

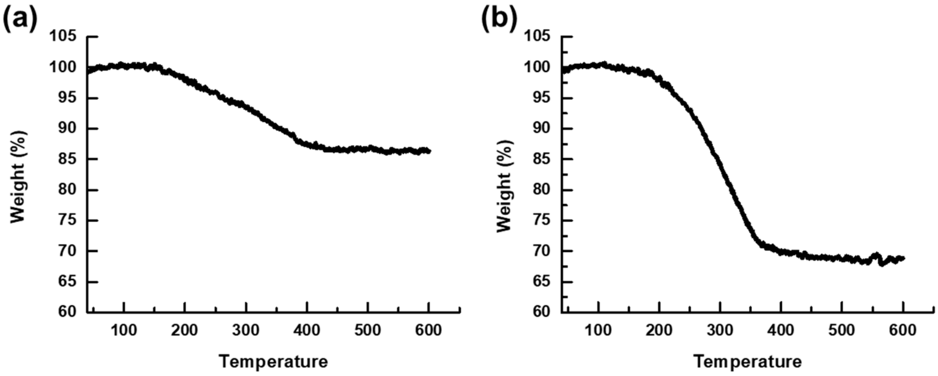

Figure S2. TGA traces of (a) Au NPs and (b) Fe_3O_4 NPs.

Table S1. Physical properties of Au and Fe_3O_4 nanoparticles.

	d_{core} (nm)	$d_{(core+shell)}$ (nm) [a]	W_L [b]	σ (chains/nm^2) [c]
Au	4.8 ± 0.5	8.1	0.14	6.02
Fe_3O_4	5.3 ± 0.5	8.2	0.32	4.56

[a] $d_{core+shell}$ was calculated by $d_{core} \times ((\frac{\rho \times W_L}{\rho_L \times (1-W_L)}) + 1)^{1/3}$; [b] Based on the weight loss measured in TGA. [c] Grafting density (σ) of the ligand was calculated based on following equation:[1-3]

$$\sigma = \frac{W_L \times N_A \times \rho \times d_{core}}{6 \times MW \times (1 - W_L)}$$

where W_L is weight fraction of the oleic ligands determined by TGA, N_A is the Avogadro constant, ρ is the density of metals (19.32 g/mL for Au and 5.15 g/mL for Fe_3O_4), d_{core} is the diameter of metal core and MW is the molecular weight of the ligands (i.e. 282.5 g/mol for oleic acid).

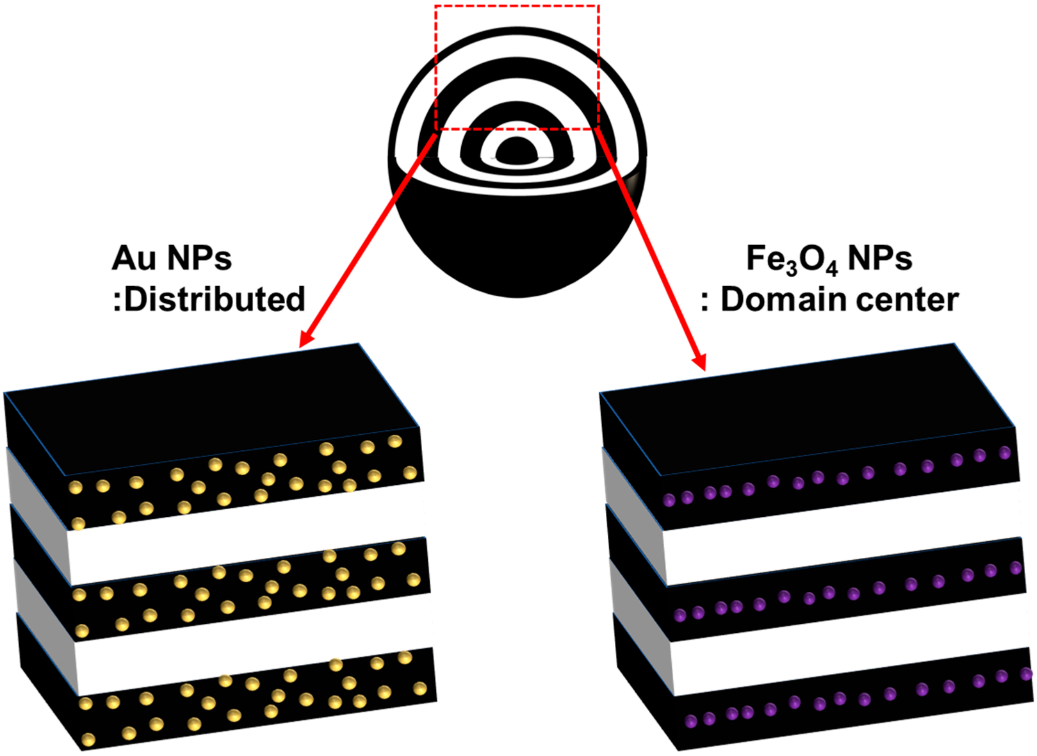

Figure S3. Schematic illustration showing the difference in the distribution of NPs inside the PB domains.

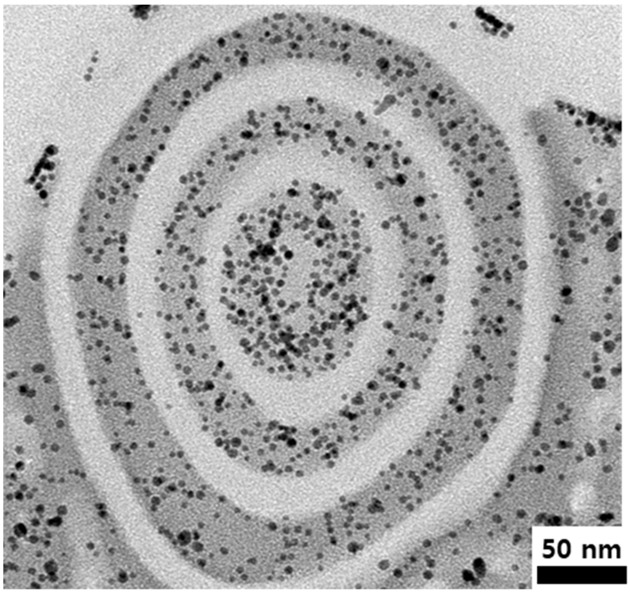

Figure S4. Cross-sectional TEM images of onion-like PS-*b*-PB/Au hybrid particles.

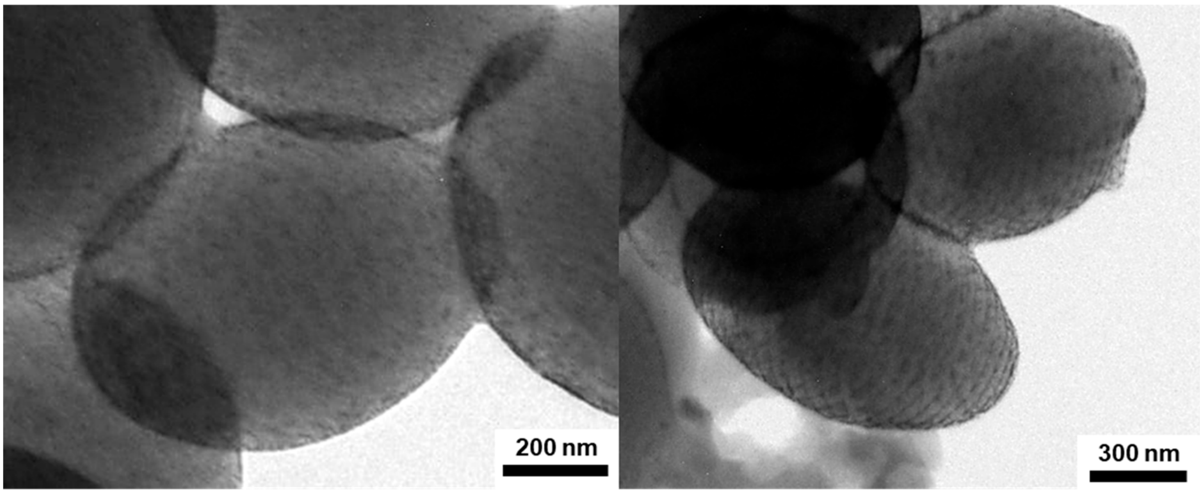

Figure S5. TEM images of tilted PS-*b*-PB/Au ellipsoids. φ = 7.0 vol%.

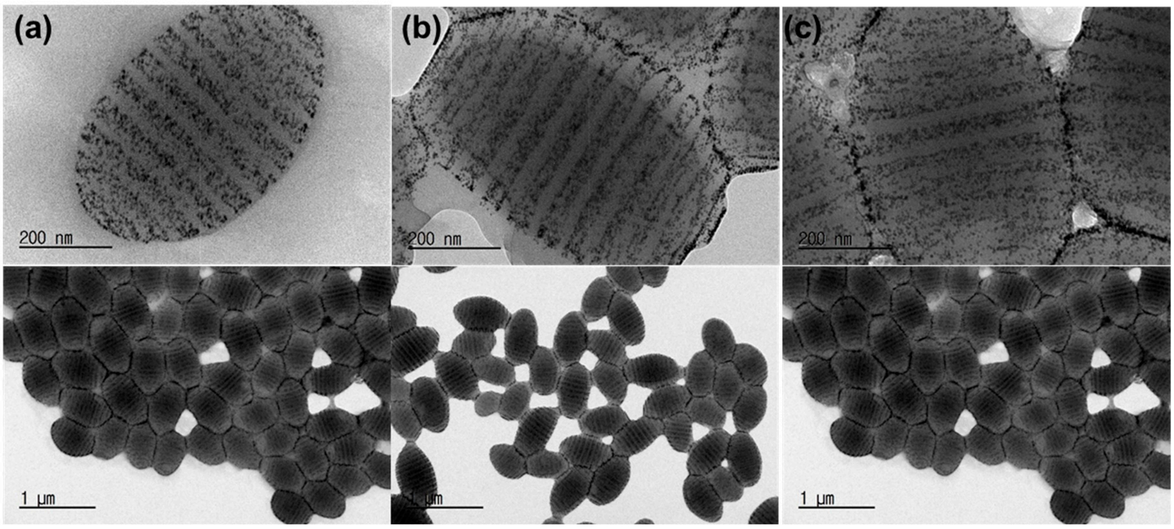

Figure S6. Magnified (upper) and low-magnification (lower) TEM images of hybrid PS-*b*-PB/Au striped ellipsoids as a function of solvent evaporation rate. (a) $A_{emul/air}$ = 26.4 cm^2, (b) $A_{emul/air}$ = 2.3 cm^2, and (c) $A_{emul/air}$ = 0.1 cm^2. φ = 7 vol%. Particles were observed without any staining.

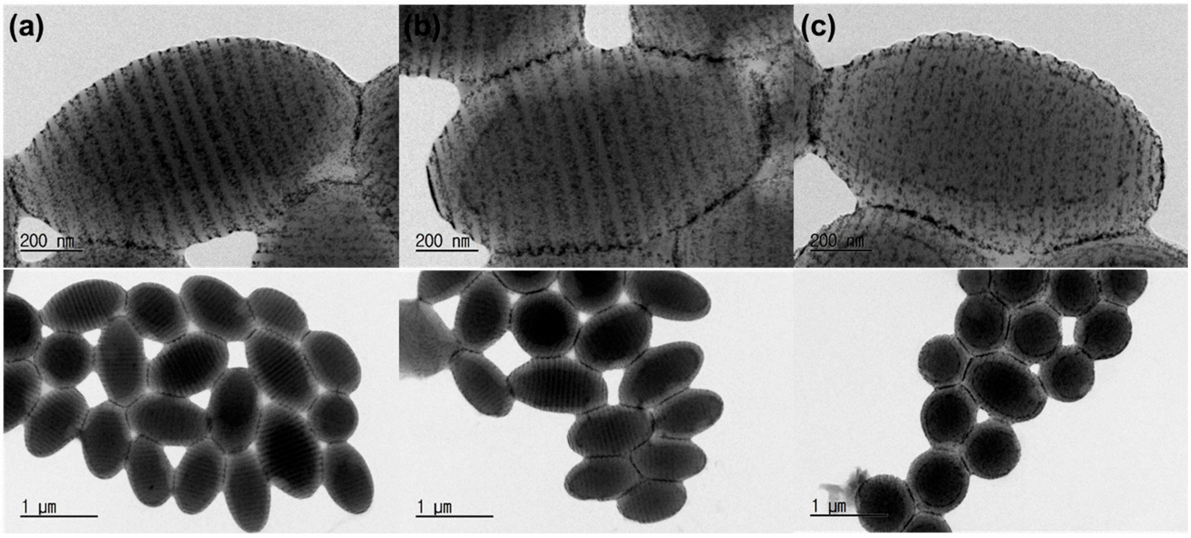

Figure S7. Magnified (upper) and low-magnification (lower) TEM images of hybrid PS-*b*-PB/Au striped ellipsoids as a function of solvent evaporation rate. Larger-sized ellipsoids were produced by using a 2.1 um SPG membrane. (a) $A_{emul/air}$ = 26.4 cm^2, (b) $A_{emul/air}$ = 2.3 cm^2, and (c) $A_{emul/air}$ = 0.1 cm^2. φ = 7 vol%. Particles were observed without any staining.

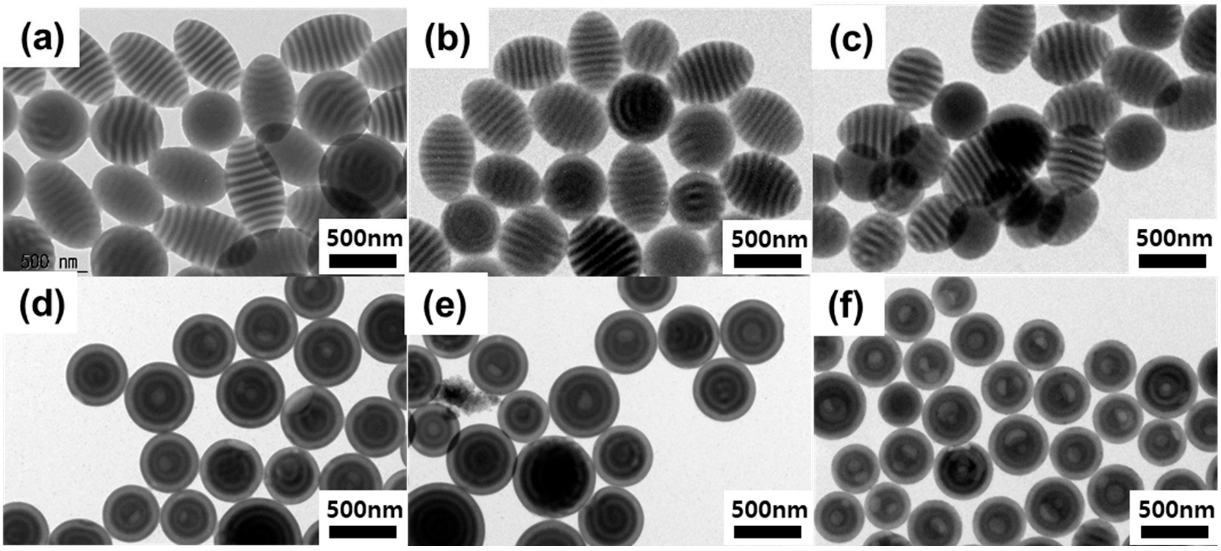

Figure S8. TEM images of hybrid PS-*b*-PB/Fe_3O_4 particles as a function of φ. (a-c) $A_{emul/air}$ = 26.4 cm^2, (d-f) $A_{emul/air}$ = 0.1 cm^2. (a, d) pristine PS-*b*-PB particles, (b, e) φ = 1.4 vol%, and (c, f) φ = 3.5 vol%. PB domains were stained with OsO_4.

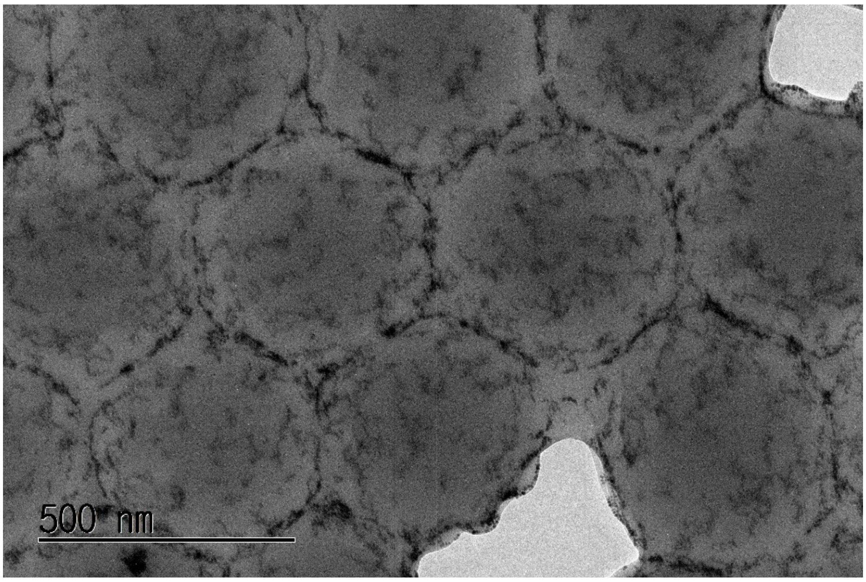

Figure S9. TEM images of hybrid PS-*b*-PB/Fe_3O_4 were obtained by freeze-drying the emulsion after evaporation of toluene for 6 hr. $A_{emul/air}$ = 26.4 cm^2.

References

1. Ye, X.; Zhu, C.; Ercius, P.; Raja, S. N.; He, B.; Jones, M. R.; Hauwiller, M. R.; Liu, Y.; Xu, T.; Alivisatos, A. P., Structural diversity in binary superlattices self-assembled from polymer-grafted nanocrystals. *Nat Commun* **2015,** *6*, 10052.
2. Xu, M.; Ku, K. H.; Lee, Y. J.; Kim, T.; Shin, J. J.; Kim, E. J.; Choi, S.-H.; Yun, H.; Kim, B. J., Effect of Polymer Ligand Conformation on the Self-assembly of block copolymers and polymer-grafted nanoparticles within an evaporative emulsion. *Macromolecules* **2021,** *54*, 3084-3092.
3. Xu, M.; Ku, K. H.; Lee, Y. J.; Shin, J. J.; Kim, E. J.; Jang, S. G.; Yun, H.; Kim, B. J., Entropy-driven assembly of nanoparticles within emulsion-evaporative block copolymer particles: crusted, seeded, and alternate-layered onions. *Chem Mater* **2020,** *32*, 7036-7043.
